# Influence of Alkali Treatment on the Microstructure and Mechanical Properties of Coir and Abaca Fibers

**DOI:** 10.3390/ma14102636

**Published:** 2021-05-18

**Authors:** Petr Valášek, Miroslav Müller, Vladimír Šleger, Viktor Kolář, Monika Hromasová, Roberto D’Amato, Alessandro Ruggiero

**Affiliations:** 1 Department of Material Science and Manufacturing Technology, Faculty of Engineering, Czech University of Life Sciences Prague, Kamycka 129, 6-Suchdol, 165 00 Prague, Czech Republic; muller@tf.czu.cz (M.M.); vkolar@tf.czu.cz (V.K.); 2 Department of Mechanical Engineering, Faculty of Engineering, Czech University of Life Sciences Prague, Kamycka 129, 6-Suchdol, 165 00 Prague, Czech Republic; sleger@tf.czu.cz; 3 Department of Electrical Engineering and Automation, Faculty of Engineering, Czech University of Life Sciences Prague, Kamycka 129, 6-Suchdol, 165 00 Prague, Czech Republic; hromasova@tf.czu.cz; 4 Departamento de Ingeniería Mecánica, Química y Diseño Industrial, E.T.S. de Ingeniería y Diseño Industrial—Technical University of Madrid (UPM), C/Ronda de Valencia, nº 3, 28012 Madrid, Spain; r.damato@upm.es; 5 Department of Industrial Engineering, University of Salerno, Via Giovanni Paolo II, nº 132, 84084 Fisciano, Italy; ruggiero@unisa.it

**Keywords:** abaca, coir, alkali treatment of fibers, tensile properties, vegetable fibers

## Abstract

Composite materials with natural fillers have been increasingly used as an alternative to synthetically produced materials. This trend is visible from a representation of polymeric composites with natural cellulose fibers in the automotive industry of the European Union. This trend is entirely logical, owing to a preference for renewable resources. The experimental program itself follows pronounced hypotheses and focuses on a description of the mechanical properties of untreated and alkali-treated natural vegetable fibers, coconut and abaca fibers. These fibers have great potential for use in composite materials. The results and discussion sections contribute to an introduction of an individual methodology for mechanical property assessment of cellulose fibers, and allows for a clear definition of an optimal process of alkalization dependent on the content of hemicellulose and lignin in vegetable fibers. The aim of this research was to investigate the influence of alkali treatment on the surface microstructure and tensile properties of coir and abaca fibers. These fibers were immersed into a 5% solution of NaOH at laboratory temperature for a time interval of 30 min, 1 h, 2 h, 3 h, 6 h, 12 h, 24 h, and 48 h, rinsed and dried. The fiber surface microstructures before and after the alkali treatment were evaluated by SEM (scanning electron microscopy). SEM analysis showed that the alkali treatment in the NaOH solution led to a gradual connective material removal from the fiber surface. The effect of the alkali is evident from the visible changes on the surface of the fibers.

## 1. Introduction

The utilization of natural fibers has been prevalent in applications within composite materials, primarily in and the textile industry. Natural fibers can come from seeds, fruits (cotton, coconut, etc.), leaves (agave, pineapple, aloe, sisal, hemp, flax, etc.) and stems (bamboo, flax, hemp, jute, kenaf, etc.). Length and thickness are given namely by the type of plant, growing conditions, etc., as opposed to synthetic fibers. It follows that the dimensions of natural fibers cannot be directly influenced. In natural fibers, their specifics, such as the structure and anisotropy of their physical and mechanical properties, need to be taken into consideration [1,2].

The effectivity of various fiber treatments used for the optimization of composite systems’ interfacial interaction (e.g., alkali treatment) can be assessed by a comparison of the tensile strength value, before and after treatment. This approach, common in engineering, is valid to a certain extent even at very small dimensions, and thus it may also be applied to human-made fibers (glass, aramid, and carbon) as a reinforcement of composites [3,4,5]. 

The current tendency to increase a portion of products from renewable resources leads to an effort to substitute synthetic fibers with natural ones. As many authors mention, natural fibers have many advantages such as their low mass (density) and their price [3,6,7,8,9,10,11,12,13,14,15]. The mechanical properties of fibers influence the resultant quality of composites, making it necessary to determine and optimize these properties. However, a determination of tensile strength is a problem in natural vegetable fibers, since published values are highly scattered, and insufficient descriptions of the resultant mechanical characteristics of arising biocomposites makes their application difficult [6,8,9,16,17,18,19,20,21,22,23,24,25,26]. 

Authors state the following tensile strengths in abaca fiber bundles: 755–798 MPa [6], 850–1400 MPa [8], 755 ± 90 MPa [9], 400 MPa [20], 452 ± 34 MPa [22], 529–754 [24], 600 MPa [26], 813 ± 34 MPa [18], and 946 ± 300 MPa [19]. We can observe similar results in coir fiber bundles, where tensile strength values have been found as follows: 131–343 MPa [6], 220 MPa [8], 175 MPa [20], 95–118 MPa [21], 137 ± 11 MPa [22], 95–230 MPa [23], 105–175 MPa [25], 112–161 MPa [16], 118–143 MPa [16], 131–175 MPa [17], 186–343 MPa [16] and 175–220 MPa [27].

**Figure 1:**
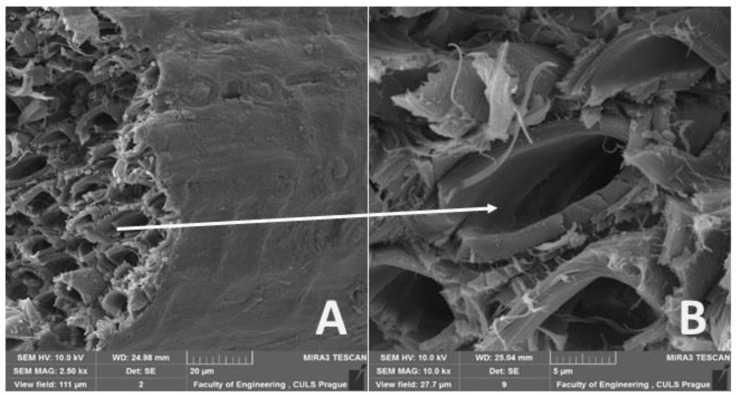
SEM images of cut through coir (**A**): Overall view of fibers (MAG 2.50 kx) (**B**): Detailed view of the fiber cut, with a polygonal shape (MAG 10.00 kx).

A comparison of the measurement results of different authors is difficult for three reasons. Setting a cross-section size of the sample for a calculation of tensile strength is a problem. Figure 1 shows a bundle of natural fibers used as a test sample, whose cross-section is of an irregular shape and contains pores.

The procedure of the cross-section area calculation is not standardized. Some publications do not even mention how the cross-section area was determined [12,28,29,30]. In most cases, a fully circular cross-section of a bundle calculated from a transverse dimension is supposed [15,16,18,19,31,32]. Munawar [22] determined the transverse dimension using five values measured while rotating the sample around a longitudinal axis by 36°. Rao [13] used four values after 45°. On the contrary, Silva [14] regarded a circular cross-section as unsuitable and determined the cross-section area by means of scanning electron microscopy (SEM). Nam [25], Yan [23], and Cai [9,33] considered a full elliptical cross-section, whose area was calculated from two measured perpendicular dimensions. Nitta [34] replaced a real cross-section area by a hexagon and calculated the area from three dimensions by rotating the sample by 60°. Sghaier [35] and Nechwatal [36] determined the cross-section area by a proportion of the fiber volume and the length. Haag [37] proved that the calculated tensile strength value predominantly depends on the method and accuracy of cross-section area determination. The strength of the same fiber bundle often differs. Results found by different methods cannot be compared. Bourmaud [6] tested four different methods of sample cross-section determination and recommended using other methods of cross-sectional area calculation before and after an alkali treatment (a change in the cross-section shape occurs). The cross-section area can be found precisely thanks to SEM technology, but the device is expensive, and the procedure is lengthy at higher amounts of samples. 

A second complication is caused by the fact that the tensile strength is not constant, even with the same type of natural fiber (the same “material”), but this depends on the transverse dimension of the fiber. This situation is caused by the fact that a full cross-section of the fiber is usually assumed at the tensile strength calculation. Lumens are not considered. Many authors, e.g., Valášek [38,39,40], have dealt with their descriptions, e.g., by porosimetry. The cross-section is porous, and fibers of different thicknesses have varying levels of porosity. A thicker fiber is of a smaller value of the portion “cell walls/lumens”, the fiber is of lower density, and the cross sections contain a lower percentage of material [22,41]. In contrast with thinner fibers, a thicker fiber is of lower strength [10,11,16,18,23]. Natural fibers of a certain thickness can have different levels of porosity or different cross-section shapes. The tensile strength is therefore difficult to use for an evaluation of natural porous fibers treatment effect. The difference in tensile strength values does not have to be caused by the fiber treatment only. It can be a consequence of various cross-section dimensions of untreated fibers and fibers chosen for treatments [18]. Various groups of samples can be of different cross sections and of different strengths, even before treatments start. It is not always possible to use a number of samples such that the difference in a fiber’s cross sections are statistically insignificant.

In addition to inconsistent cross-section dimension determination procedures and the dependence of the resultant tensile strength on the tested fibers’ dimensions, the influence of locality conditions under which a plant is grown (weather, soil), the procedures used to obtain fibers from a plant, and the maturity degrees, among others, represent the third cause of the wide distribution of published values regarding the same type of fiber [8,10,11,16,42,43].

One existing approach to determining fibers’ mechanical properties which minimizes the first two mentioned problems is more suitable, especially for natural fibers with small, irregular, and porous cross sections [44]. It is commonly used in the textile industry when evaluating yarn strength [45]. The breaking tenacity f (cN/tex) is calculated instead of the tensile strength Rm (MPa). 

While it is a common method in the textile industry, when evaluating the mechanical properties (in engineering) of natural fibers determined as a reinforcement of composites, breaking tenacity is only rarely considered. For example, Negawo [46] compared untreated and alkali-treated Ensete stem fibers by means of breaking tenacity, Yilmaz [47] compared the corn husk fibers, Mylsamy [7] compared agave fibers, Abdel-Halim [48] compared linen fibers, Boopathi [49] compared borassus fruit fibers, Ray [50] and Vigneswaran [51] studied jute fibers and Reddy [52] studied cotton.

A low adhesion to a polymeric matrix prevents a wider application of natural fibers in composites. It does not come to a load transfer from the matrix to the fibers, and relatively good mechanical properties of fibers are not fully utilized [10,11,53]. Various physical and chemical treatments of fibers have been performed in order to improve adhesion. For example, George [42] and Bledzki [3] presented a detailed overview of the methods used. Alkalization, i.e., the soaking of fibers in a water solution of NaOH, is a common chemical treatment. It has been historically the oldest method used for improving the adhesion of colors to cotton fibers, and since the middle of the 19th century has been known as mercerization [7].

From a chemical point of view, natural vegetable fibers above all consist of cellulose, hemicellulose, and lignin. The rest is pectin, wax, and other surface impurities. Various types of fibers are comprised of various ratios of these components [10,27]. Cellulose molecular chains mutually connected by hydrogen bonds are in the form of microfibers [54], which are bound to each other by hemicellulose and lignin, and create elementary fibers and bundles [11,25]. The mechanical properties of natural fibers depend on the ratio size of individual components [3,6,10]. Free hydroxyl groups of cellulose OH^-^ cause an undesirable hydrophilic nature in fibers [10,20,43]. Owing to a different solubility of individual fiber components in an NaOH solution and an ability of NaOH to react with a hydroxyl group, the presented properties can be changed by the alkalization. Cellulose is almost insoluble, while on the contrary, hemicellulose dissolves easily. Lignin is soluble only to a limited extent, a higher concentration and temperature of the NaOH solution helps to improve solubility [10,20]. 

A double effect is reached by a dissolution of the surface lignin. The fiber surface is roughened, and a reaction of exposed cellulose hydroxyl groups is simultaneously enabled to a greater extent [11,27,31]. Owing to this crosslinking of cellulose, molecular chains and the microfiber strength are increased. Hydrogen atoms in the free hydroxyl groups are replaced by sodium atoms [20]. Higher adhesion between matrices and fibers is reached by this. A reduction of the fiber cross-section (shrinkage) is observed simultaneously due to an extinction of cavities [9,33,55], and thus the effective contact surface of the fibers in composites is increased [56]. The often-published increase in the fiber’s theoretical tensile strength owing to alkalization [9,12,25,28,32,33,49,57] can be, in accordance with the definition, partly caused by the reduction of the cross-section and by the reduction of the porosity without a loss of bearing cellulose.

If enough lignin is dissolved, the bundles are disintegrated; on the contrary, contact will worsen due to a limited run-in of the matrix among the individual fibers [33]. An excessive action of NaOH may result in a deterioration of mechanical properties, not only of composites but also of individual fibers [11,12,19,27,30,35,43,48].

Based on the above, it is obvious that it is necessary to determine a suitable concentration, eventually a temperature, and an optimal time for fiber immersion into an NaOH solution, which can be different for each fiber type. Data of various authors regarding suitable parameters differ substantially. Symington [19] summarized data that have been found. Alkali treatment should not lead to an essential deterioration of fiber mechanical properties. It was found that, by studying references, a certain correlation between the lignin content in natural fibers and optimal alkali parameters exists. More lignin causes a longer action time, and a higher concentration or temperature of the NaOH solution is eventually necessary.

The experimental part of this paper determines the mechanical properties of untreated and alkali-treated natural vegetable fibers. The alkalization of cellulose fibers was chosen for the experimental program because it is a simple and verified method of chemical treatment. 

Measured results were evaluated by calculation of the tensile strength tenacity, which was analyzed against the literary background and allowed for a definition of an optimal methodologic procedure for determining the mechanical characteristics of cellulose fibers. 

The aim of this research was to investigate the influence of alkali treatment on the surface microstructure and tensile properties of coir and abaca fibers. The paper presents results focused on the relation between the surface microstructure of fibers and the tensile properties depending on different time intervals of 5% NaOH solution treatment. The results of the SEM analysis of fiber surfaces, comparing the mechanical properties of untreated fibers (marked as 0 h treatment) and alkali-treated fibers by immersing them into a 5% NaOH solution at laboratory temperature for different time intervals from 0.5 h, support their utilization for the surface modification of natural coir and abaca fibers as composite materials. 

Coconut fibers are obtained from coconut bark growing on palm trees. Coconut fibers are natural vegetable fibers with the highest content of lignin and the lowest content of hemicellulose. Rout [53] states that coconut fiber properties cannot be compared with other types of fibers owing to their composition. Abaca fibers or Manila cannabis (occurring mainly in the Philippines) are obtained from leaves of the plant Musa textiles. Abaca fibers, by contrast, have a relatively low content of lignin. 

The experiment results have an unambiguous influence on defining a united optimal methodology for determining the mechanical properties of fibers that are presently used as filler in modern composite materials based on renewable sources.

## 2. Materials and Methods 

Fibers with different cellulose, hemicellulose, and lignin contents were chosen for the experimental program due to an effort to describe the differences in behavior in alkali treatment. Brown coir fibers with a cellulose content of 32–47% and a lignin content of 40–46% and abaca fibers with a cellulose content of 56–68% and a lignin content of 9–15% were tested [58]. Fibers were obtained from the processing of plants in the Philippines. Only fibers with a certain interval of means, i.e., from 180 to 280 μm, were chosen. Fibers with very low and high mean values were eliminated from the measurements to eliminate their influence on the measured values. The mean values were determined on the stereoscopic microscope Zeiss Stemi 508 with an Axiocam and evaluated by software. These values were not entered into calculations.

### Alkalization

Fibers were weighed with an accuracy of 0.1 mg on Kern APS 120–4 scales (accurate measurements were used, and since values may be affected by the moisture content of the fibers caused by the humidity of the laboratory environment, fibers were dried before measurement). Their lengths were also measured. The linear density of the fibers in mg/m (tex according to the textile industry) was calculated from the weight and height. This measurement was performed before and after treatment in a 5% solution of NaOH.

Fibers were individually immersed into the 5% solution of NaOH at a laboratory temperature at the time intervals 30 min, 1 h, 2 h, 3 h, 6 h, 12 h, 24 h, and 48 h. Each group was created from 10 fibers. Fibers exposed to the 5% solution of NaOH were subsequently transferred to a beaker with distilled water for rinsing. Fibers were dried at the laboratory temperature. Subsequently, fibers were glued with gel instant adhesive to cardboard, according to the standard EN ISO 5079:1997 [59]. The fiber length for the test was 10 mm. The measurement was performed after 24 h on the universal testing machine Labortech, type MP Test 5.050, scanner KAF-S (0–5000 N), with a load cell in a measuring range from 0 to 50 N. The fiber with the cardboard was clamped between rubber jaws at a distance of 10 mm, and the cardboard was cut. The tensile test speed was 1 mm/min. The course of the force depending on the crossbar position, the force itself, and the elongation at the fiber rupture were recorded. The cardboard pieces with the fiber were placed into marked bags after the test. From the force course depending on the crossbar position, the dependence of the tension on the elongation was calculated, and Young’s modulus was determined as the slope of the initial straight line. The tensile strength and the elongation at break were calculated from the maximum force. 

The transverse dimension of individual fibers was measured using the stereoscopic microscope Zeiss Stemi 508. Three measurements were made on both ends of the fiber near the rupture location. The average value from six measured transverse dimensions was used for the calculation of tensile strength. A fully circular cross-section of the fiber was assumed and used as the standard dimension for the calculated characteristics. 

The evaluation of measured data was performed by means of the program STATISTICA, by an ANOVA F-test, i.e., the hypothesis H_0_ presents a statistically insignificant difference among the measured data (*p* > 0.05), and the hypothesis H_1_ presents a refusal of the hypothesis H_0_, i.e., there is a statistically significant difference among the measured data (*p* < 0.05) [60].

## 3. Results and Discussion

This research focused on the evaluation of mechanical properties and the structure of alkali-treated coir and abaca fibers in a 5% solution of NaOH. The alkali treatment of the fibers leads to a gradual removal of binding materials [9].

Natural fibers show poor compatibility with polymeric matrices and a relatively high absorption of the humidity. These properties of natural fibers influence the resultant properties of the composites [27,61]. In order to minimize these undesirable properties and to improve the adhesion, it is necessary to perform the chemical treatment of natural fibers [61].

**Figure 2:**
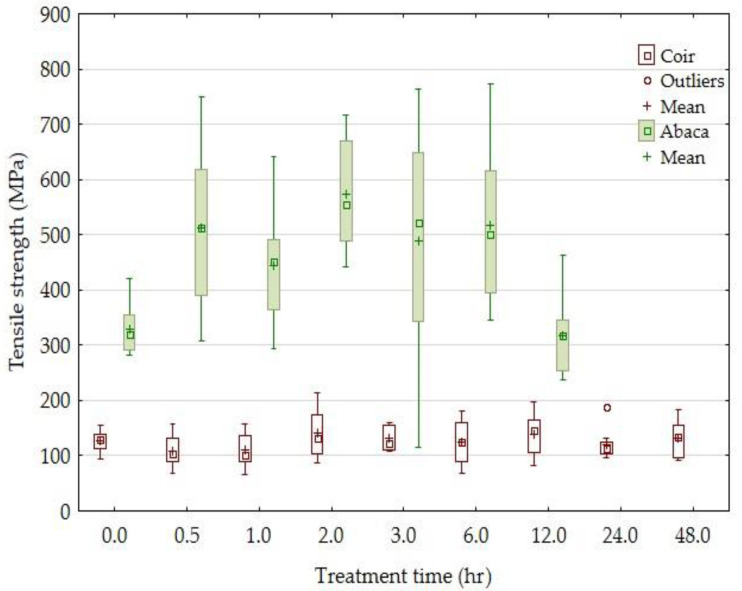
The dependence of the tensile strength of the coir and abaca fibers on the time of treatment in a 5% solution of NaOH.

**Figure 3:**
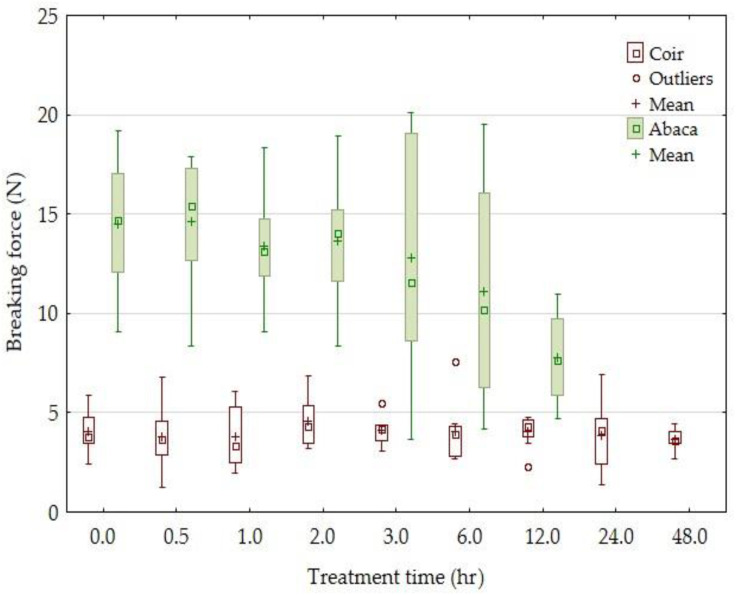
The dependence of the breaking force of the coir and abaca fibers on the time of treatment in a 5% solution of NaOH.

**Figure 4:**
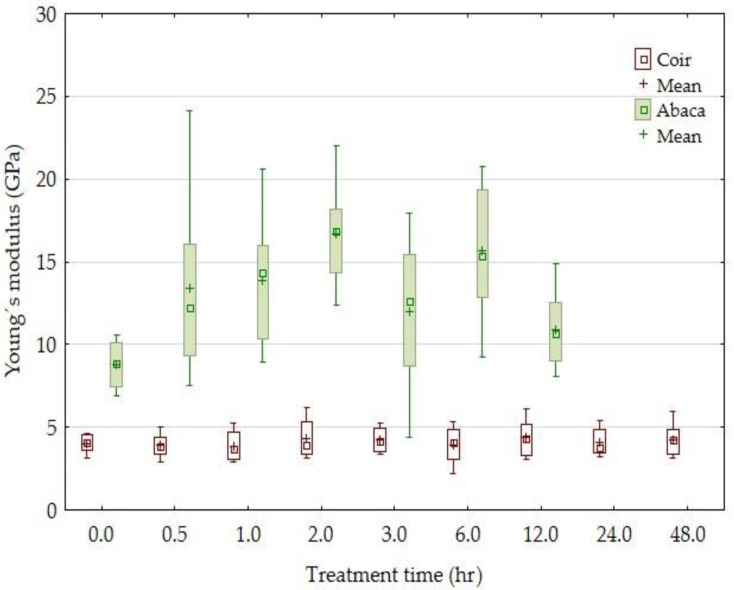
The dependence of the Young’s modulus of the coir and abaca fibers on the time of treatment in a 5% solution of NaOH.

**Figure 5:**
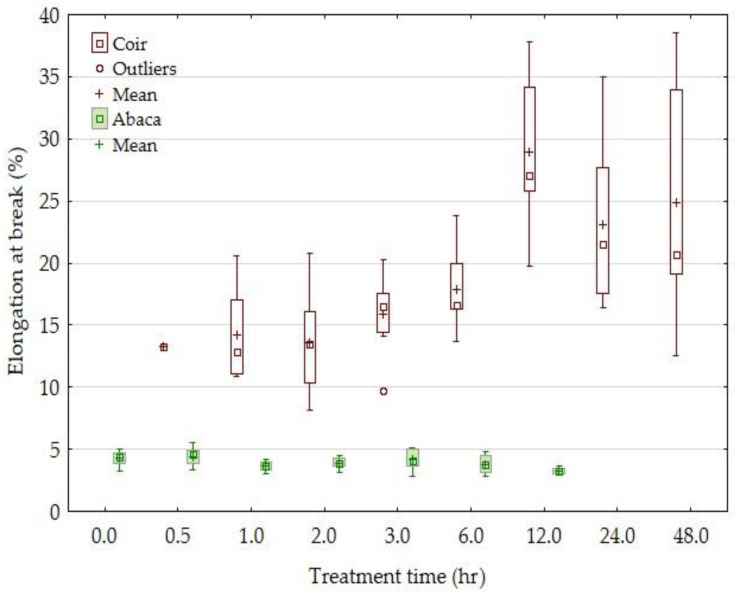
The dependence of the elongation at break of the coir and abaca fibers on the time of treatment in a 5% solution of NaOH.

Results of the tensile strength of the tested fibers and the influence of the alkali treatment time are compared in Figure 2. Results of the experiments show (Figure 2, Figure 3, Figure 4 and Figure 5) the change in the mechanical properties: the tensile strength, breaking force (the force measured during the destruction of the test sample), and Young’s modulus of the abaca fibers treated at different time intervals in a 5% solution of NaOH. The tensile strength of the abaca fibers was increasing in the interval from 0 to 6 h up of 74%. Subsequently, a gradual decrease in the tensile strength occurred. A similar course of experiments was also observed in terms of Young’s modulus, i.e., it was increasing from 0 to 6 h up of 90%. Subsequently, a gradual decrease in Young’s modulus occurred.

Negawo [46] determined the optimal time of abaca treating in a 5% solution of NaOH to be 2 h. A similar result is evident from results presented in Figure 2.

Experiment results also show (Figure 2, Figure 3, Figure 4 and Figure 5) mild changes in the mechanical properties: the tensile strength, breaking force, and Young’s modulus of the coir fibers treated in a 5% solution of NaOH for the interval from 0 to 48 h. A significant change in the mechanical properties did not occur at the tested coir fibers in the tested time interval from 30 min to 48 h.

The results of statistical testing, i.e., the values of the parameter *p*, are shown in Table 1.

One difference among the results of mechanical tests was found in the influence of the time interval in the 5% solution of NaOH. The differences in treatments of coir and abaca fibers were caused by different concentrations of lignin. The abaca fibers contained a lower percentage of lignin (around 9%), while the coir fibers contained 40–46% [58]. The dissolution of the surface lignin and the crosslinking of cellulose molecular chains result in an increase in the microfiber strength [11,27,62]. However, a substantial amount of lignin is dissolved after a long period of time. This state can be determined by the disintegration of elementary fibers (fibril bundles) leading to the deterioration of mechanical properties [11,12,19,27,30,33,35,43,48]. This state manifested in abaca fibers after 12 h in the 5% solution of NaOH. A lower content of lignin also increases the speed of its solubility. This undesirable state was proved by SEM images.

Monteiro [63] states that the alkali treatment of the coir does not work even after a long period of time owing to the improvement in the mechanical properties. However, Kabir [43] states that the fiber’s mechanical properties depend significantly on the maturity degree. Therefore, the results of mechanical tests can differ among different authors. The tensile strength of the abaca fibers differed in this experiment, i.e., it was smaller than is presented in the available literature [6,8,9,18,19,20,22,24,26]. This can be caused by the fact that fibers with a higher cross-section were chosen for the experiment. A smaller cross-section of the fibers would increase the strength. The tensile strength results of the coir fibers are in a good correlation with the published results of other authors [6,8,16,20,21,22,23,25,27].

Although the alkali treatment does not improve the mechanical properties of the coir, it improves the interfacial interface between a matrix and a composite [33,64,65]. The properties are improved after a longer period of time in the coir, owing to a high content of insoluble lignin [25,28]. Biological fibers are suitable for application in composites due to their strength [66].

Some researchers such as Sari [57], Yilmaz [47], Negawo [46], Mylsamy [7], and Richter [26] have used tensile strength and breaking tenacity for the evaluation of fiber strength.

It is clear from Figure 3 that the force values did not change much owing to the alkali treatment time. A considerable change occurred in the abaca fibers only in the 5% solution of NaOH acting for 12 h.

The results of the elongation at break are evident in Figure 5. The elongation at break was considerably lower in the tested abaca fibers than in the coir fibers. The elongation at break of the abaca fibers was in the interval 3.25–4.26% and was decreased by the alkali treatment. On the contrary, the elongation at break of the coir was in the interval 13.3–28.5% and was considerably increased by the alkali treatment. The results are in good correlation with the results of Militký [58], who stated the elongation at break of the coir was 15–40% and that of the abaca fibers was 2–3%. The elongation at break of the coir is highest in cellulose fibers [58]. The relatively high elongation at break of the coir was caused by a high microfibrilar angle. This is a tilt of the cellulose microfiber from the fiber axis. For destruction, it is necessary to straighten the cellulose microfiber to the fiber axis.

The alkali treatment leads to a removal of hydrogen bindings in the crosslinked networks of the cellulose and lignin structures [11,29], and the fibers become “soft” by this [67], which leads to a high elongation and a decrease in Young’s modulus [29]. 

The research results of the abaca fibers proved statistically significant differences in the tested parameters of the mechanical tests (tensile strength, breaking force, elongation at break, and Young’s modulus) depending on the alkali treatment time, i.e., the chemical action of the 5% solution of NaOH on the mechanical properties of the abaca fibers occurred in the interval from 0 to 12 h.

The statistically significant difference in the tested mechanical parameters (tensile strength, breaking force, and Young’s modulus) was not proved with the tested coir fibers owing to the alkali treatment time, i.e., the chemical action of the 5% solution of NaOH on the mechanical properties of the coir fibers occurred in the interval from 0 to 48 h, except for the elongation at break, where a statistically significant difference depending on the alkali treatment time was proved.

**Figure 6:**
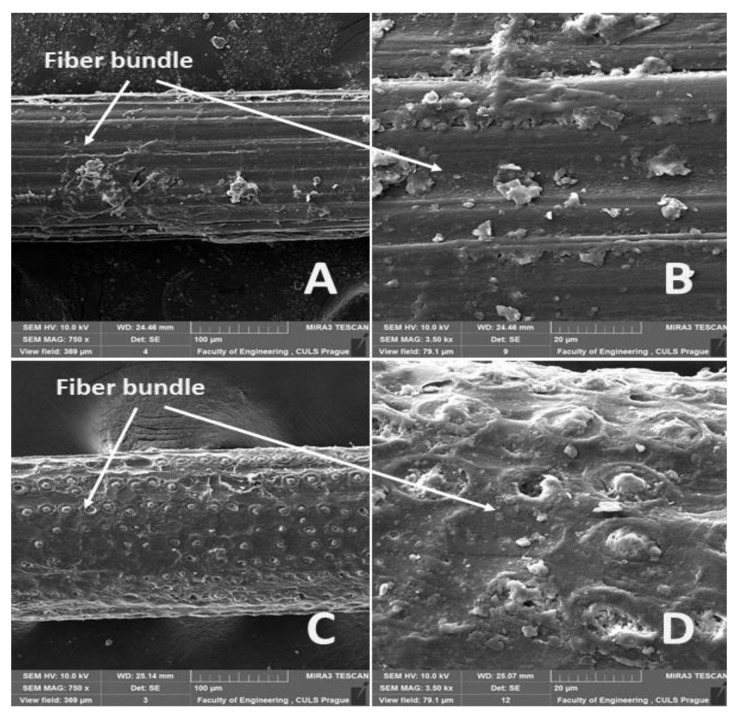
SEM images of the surfaces of abaca and coir fibers without treatment in a 5% solution of NaOH (**A**): Abaca fiber bundle (MAG 750 x) (**B)**: detailed view of the surface of an abaca fiber (MAG 3.50 kx), (**C**): coir bundle (MAG 750 x), (**D**): detailed view of the surface of a coir fiber (MAG 3.50 kx).

**Figure 7:**
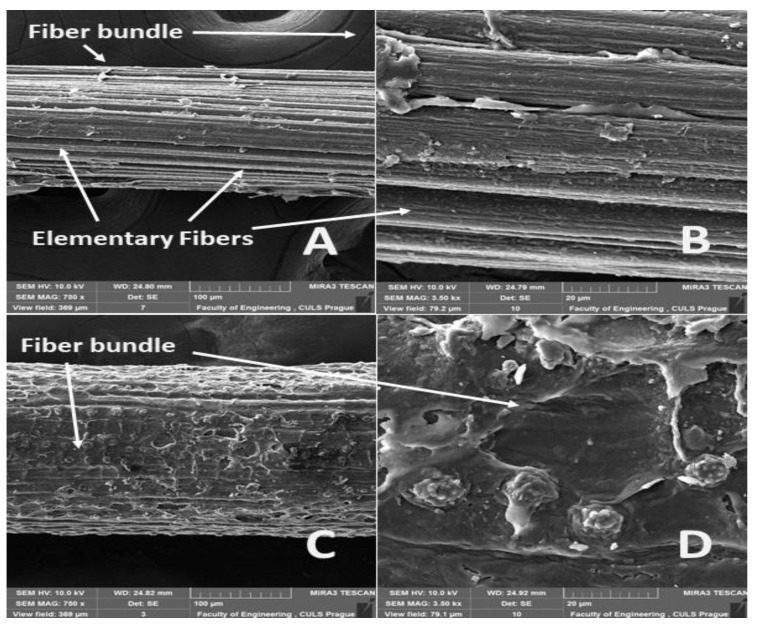
SEM images of the surfaces of abaca and coir fibers with treatment in a 5% solution of NaOH for 1 h (**A**): abaca fiber bundle (MAG 750 x), (**B**): detailed view of the surface of an abaca fiber (MAG 3.50 kx), (**C**): coir bundle (MAG 750 x), (**D**): detailed view of the surface of a coir fiber (MAG 3.50 kx).

**Figure 8:**
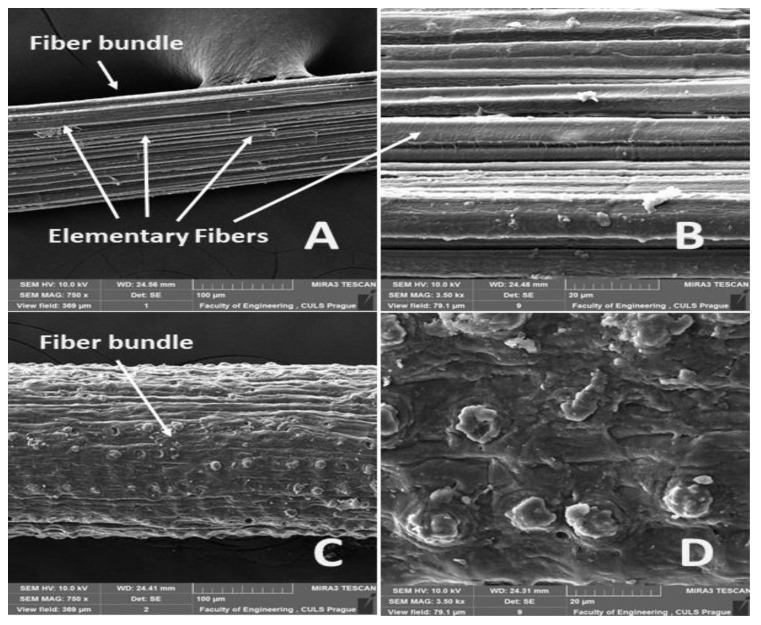
SEM images of the surfaces of abaca and coir fibers with treatment in a 5% solution of NaOH for 6 h: (**A**): abaca fiber bundle (MAG 750 x), (**B**): detailed view of the surface of an abaca fiber (MAG 3.50 kx), (**C**): coir bundle (MAG 750 x), (**D**): detailed view of the surface of a coir fiber (MAG 3.50 kx).

**Figure 9:**
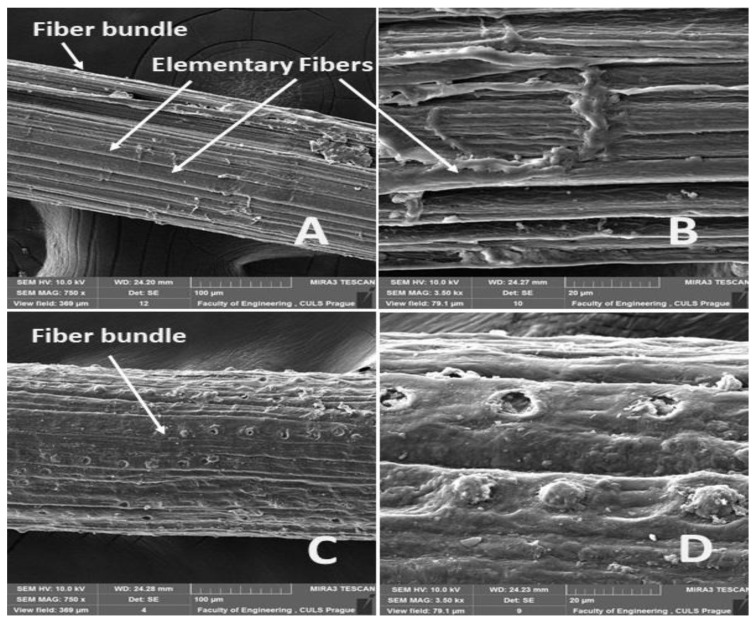
SEM images of the surfaces of abaca and coir fibers with treatment in a 5% solution of NaOH for 12 h: (**A**): abaca fiber bundle (MAG 750 x), (**B**): detailed view of the surface of an abaca fiber (MAG 3.50 kx), (**C**): coir bundle (MAG 750 x), (**D**): detailed view of the surface of a coir fiber (MAG 3.50 kx).

**Figure 10:**
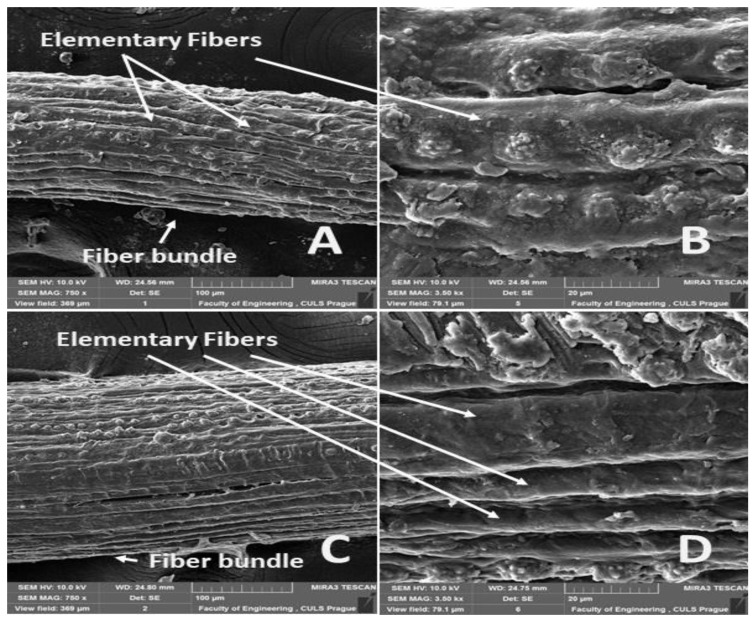
SEM images of the surfaces of coir with treatment in a 5% solution of NaOH: (**A**): coir bundle treated in the 5% solution of NaOH for 24 h (MAG 750 x), (**B**): detailed view of the surface of the coir treated in the 5% solution of NaOH for 24 h (MAG 3.50 kx), (**C**): coir bundle treated in the 5% solution of NaOH for 48 h (MAG 750 x), (**D**): detailed view of the surface of the coir treated in the 5% solution of NaOH for 48 h (MAG 3.50 kx).

The surfaces of the abaca fiber and the coir without and with alkali treatment in the 5% solution of NaOH at different time intervals are evident from Figure 6, Figure 7, Figure 8, Figure 9 and Figure 10. The figures show the microstructure of the fiber surface where changes are clearly observable. The surface of the untreated natural fibers represents undesirable globular protrusions at irregular intervals [27]. The alkali treatment of the natural fibers seems to be effective at removing undesirable residues from the surface, as is evident from SEM research [27]. This conclusion was also proved in a study of chemical composition [27].

The alkali treatment removes surface binding materials from the fiber bundles. The surface lignin was dissolved by the NaOH solution acting distinctly for a double effect, shown in Figure 7, Figure 8, Figure 9 and Figure 10. Natural fiber processing (banana fibers) by means of NaOH effectively removed non-cellulose materials and other impurities from fiber surfaces, and the surface was smooth [68]. The banana fiber surface had more perceptible pores than the fibers treated in NaOH [68]. The SEM analysis of the natural fibers (banana) revealed that the surface impurities were removed during NaOH treatment [68]. It was also proved that the surface area of NaOH-treated banana fibers was improved, which can lead to better interfacial properties of fibers [68].

The first effect is a fiber surface roughening and a subsequent fiber bundle disintegration.

Similar conclusions were proved in a paper entitled “Characterization of cellulose fibers in Thespesia populnea barks: Influence of alkali treatment”, where the authors state that alkali treatment causes a rougher surface and helps to disintegrate the fiber surface [69]. Senthamaraikannan and Kathiresan [70] also addressed the subsequent surface roughness of treated fibers. The surface roughness parameters Ra were influenced by the alkali effect on hemicellulose, lignin and other impurity removals [70].

The significantly faster dissolution of the surface lignin and the connected disintegration of the fiber bundles occurred at the abaca fibers (Figure 7, Figure 8 and Figure 9A,B). Cai [33] pointed out this negative state in his study. One possible explanation is, as Kalia [11] states, that the binding materials, such as pectin, lignin, and hemicellulose, were removed from the abaca fibers during the alkali treatment, which led to the fibrillation and disintegration of the fiber bundles into the elementary fibers.

Authors have found that the surface roughness of natural fibers after the alkali treatment increases with the disintegration of a protective hemicellulose and lignin structure and a waxy structure during alkali treatment [70,71]. They expected that this would lead to an increase in the contact between the real surface area and the environment, and that this would improve the adhesive interface between fibers and the polymer in the composites [70,71].

The second effect is the dissolution of the surface lignin.

The reaction of the uncovered hydroxyl groups of the cellulose is simultaneously enabled to a greater extent at the fiber surface roughening [11,27,62]. The crosslinking of the cellulose molecular chains increases owing to this, which increases the microfiber strength. It is important to determine the optimal time, which differs depending on the fiber. For example, shorter times of alkali treatment are necessary for abaca fibers, as opposed to coir. The reason for this is that the excessive action of NaOH may result in the deterioration of the mechanical properties of individual fibers [11,12,19,27,30,35,43,48]. It is clear in Figure 10A,C that the coir was twisted in the longitudinal direction after 24 h and 48 h in the 5% solution of NaOH, but the fibers bundles were not considerably disintegrated. There is a presumption for the fiber utilization in polymer composite materials that the fiber bundle should be shrunk and that the surface should be roughened but not divided into individual fibers.

**Figure 11:**
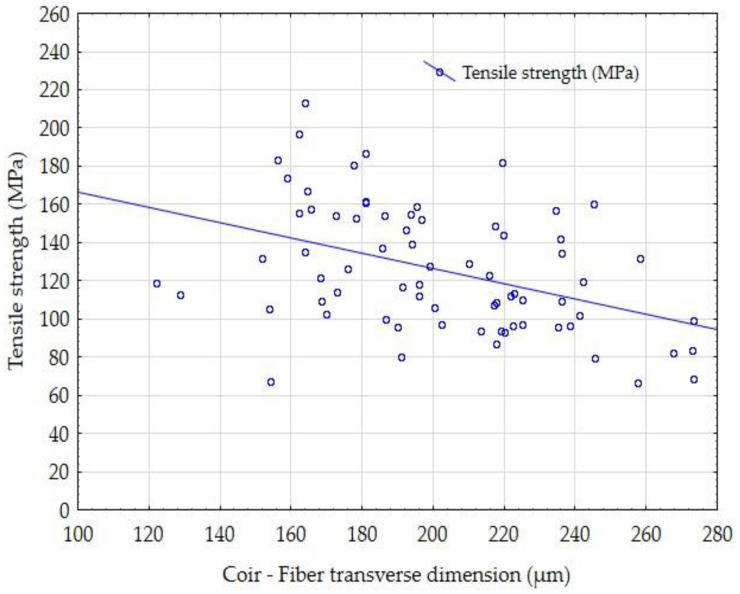
Comparison of the influence of the fiber transverse dimension of the coir on the tensile strength.

**Figure 12:**
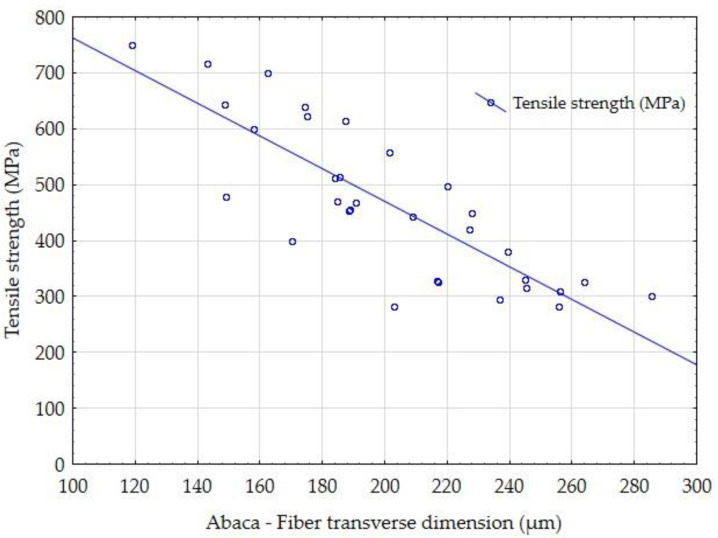
Comparison of the influence of the fiber transverse dimension of the abaca fibers on the tensile strength.

The influence of the fiber transverse dimension of the tested coir and abaca fibers on the tensile strength is shown in Figure 11 and Figure 12. It is obvious that the tensile strength of the fibers depends on the dimension. The tensile strength decreases as the transverse dimension of the fibers increases. This trend is evident in the results of the abaca fiber’s tensile strength (Figure 12). Generally, determining the tensile strength of vegetable fibers is problematic and is connected with a high variance of results. The dependence of the resultant tensile strength on the fiber transverse dimension is one of the significant factors shown in the results of the tested fibers presented in Figure 11 and Figure 12. Mizera et al. [72] found in Ensete fiber testing that fibers showed different mechanical characteristics in different places due to the change in the fiber cross-section. The trends showed that the natural fibers approach the behavior of synthetic fibers in the observed characteristics [72]. The trend is therefore the same in terms of physical dependencies [72].

Table 2 shows the trend equations of the influence of tensile strength on the fiber transverse dimension of the tested coir and abaca fibers.

**Figure 13:**
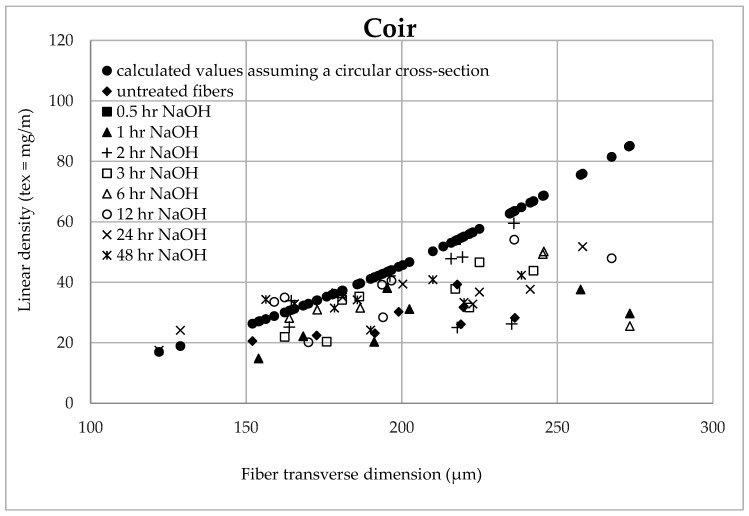
Dependence of the linear density on the transverse dimension of the coir.

**Figure 14:**
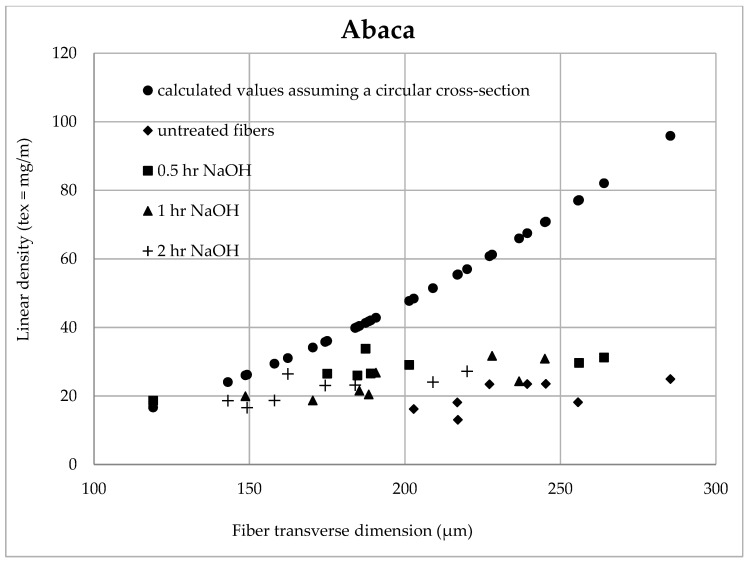
Dependence of the linear density on the transverse dimension of the abaca fiber.

Figure 13 and Figure 14 state the influence of the fiber transverse dimension on the calculated (assuming the circular cross-section) and measured linear density of the tested coir and abaca fibers. It is obvious that the theoretical (theoretically calculated) linear density is quadratically increasing (assuming a constant density or a constant porosity). The measured values of the linear density broken down into untreated fibers and fibers treated for a certain time in the 5% solution of NaOH follow a linear trend. The porosity increases as the fiber transverse dimension increases and the mass of the fiber bearing material decreases.

A different porosity of individual tested fibers is also obvious, i.e., a high scatter of values of measured masses is also in a similar transverse dimension. It is not possible to speak generally about the porosity of the specific fiber type.

Regarding abaca fibers, the results are presented until the time when the optimal treatment time in the 5% NaOH solution is reached, i.e., until 2 h, determined from information from the author Negawo [46].

It is possible that the porosity of larger fibers is increasing, i.e., the measured values of the linear density differ more from the theoretical ones (theoretically calculated). This fact results in larger errors in the tension value calculation regarding the circular cross-section.

This is a reason why the tensile strength of the fibers decreases as the fiber transverse dimension increases, i.e., there is a smaller ratio of the cell walls in the fiber cross-section.

Table 3 shows the trend equations of the influence of the fiber transverse dimension on the calculated (assuming a circular cross-section) and the measured linear density of the tested coir and abaca fibers.

**Figure 15:**
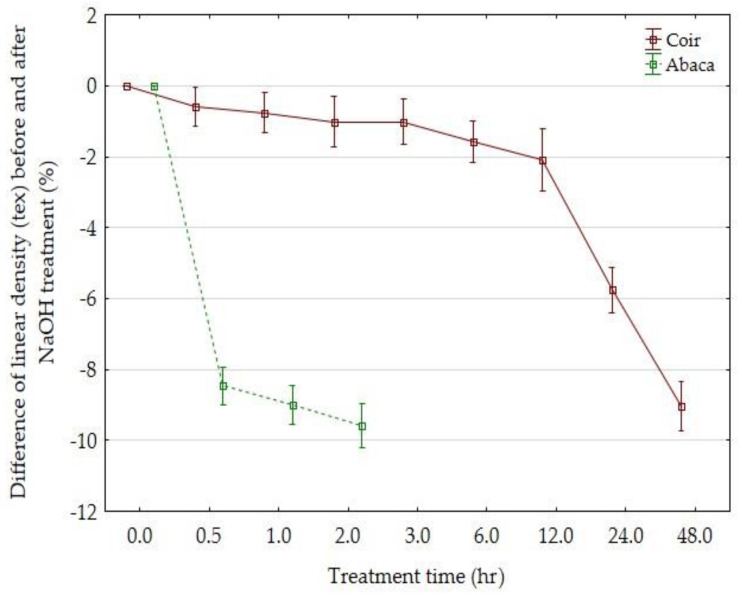
Dependence of the linear density on NaOH action.

A different dependence of the linear density of the coir and abaca fibers on the time of NaOH action is shown in Figure 15. An immediate fall in the linear density value occurred with the abaca fibers. This quick fall is caused by a high portion of hemicellulose. With the coir fibers, the fall occurs after a longer time (after 12 h). The reason is that coir has a high amount of poorly soluble lignin and a minimum amount of hemicellulose. For this reason, a longer time of action is necessary at the fiber alkali treatment. This result is also evident in SEM images that define the details of the surface and the microstructure.

## 4. Conclusions

The research results herein are focused on the evaluation possibilities of the mechanical properties of two significant biological cellulose fibers, namely coir and abaca fibers, which were alkali-treated in a 5% solution of NaOH. Before the utilization of specific biological cellulose-based fibers, it has always been necessary to measure their mechanical properties and to optimize alkali treatment parameters, namely the time of treatment and the NaOH concentration. It is not possible, for many stated reasons, to rely on published results, i.e., the measurements of other authors, although they have measured fibers of the same plant type. The results found in this study can be summarized as follows:

- Scanning Electron Microscopy (SEM) analysis defined the details of the surface and microstructure of coir and abaca fibers and showed that they do not have the same course of surface alkali treatment. Different time intervals had to be used to reach the optimal conditions of treatment, i.e., the optimal tensile properties. The alkali treatment had two effects: It roughened the fiber surface and gradually disintegrated the fiber bounds. These effects were considerably faster with abaca fibers.
- The tensile properties of the abaca fibers changed owing to the alkali treatment. The Young’s modulus increased to 90%, and the tensile strength increased to 74% compared with the abaca fibers that were not alkali-treated. The results showed only mild changes in the Young’s modulus and the tensile strength of the coir fibers. The excessive, long-term action of NaOH solution alkali treatment caused a deterioration in the mechanical properties of individual fibers.
- The tensile strength decreased as the transverse dimension of the fibers increased, namely in the abaca fibers. The porosity of the tested fibers rose as the transverse dimension of fibers increased, and the mass of the material bearing the fiber diminished simultaneously.

## Figures and Tables

**Table 1:** Statistical evaluation of static tensile test according to the ANOVA F-test with stated parameter *p* at significance level α = 0.05 at a change in the alkali treatment time in the 5% solution of NaOH.

Fiber Type	Tensile Strength (MPa)	Breaking Force (N)	Young’s Modulus (GPa)	Elongation at Break (%)
**Abaca (parameter p)**	0.0015	0.0193	0.0021	0.0056
**Coir (parameter p)**	0.5210	0.9599	0.9529	0.0000

**Table 2:** Trend equations: Influence of the tensile strength on the fiber transverse dimension of the tested coir and abaca fibers (δ—tensile strength, Tt—treatment time).

Coir	Abaca
δ = −0.4012 × Tt + 206.68	δ = −2.9178 × Tt + 1053.8

**Table 3:** Trend equations: Influence of the fiber transverse dimension on the calculated (assuming a circular cross-section) and measured linear density of the tested coir and abaca fibers (T-linear density, Ftd–fiber transverse dimension).

Treatment Time	Coir	Abaca
**Untreated**	T = 0.1450 × Ftd − 1.4440	T = 0.1072 × Ftd − 5.1974
**0.5**	T = 0.1120 × Ftd − 1.3320	T = 0.0716 × Ftd − 13.581
**1.0**	T = 0.1508 × Ftd − 0.3257	T = 0.1181 × Ftd − 0.7763
**2.0**	T = 0.1820 × Ftd − 0.8572	T = 0.1037 × Ftd − 4.1013
**3.0**	T = 0.2645 × Ftd − 19.324	–
**6.0**	T = 0.0760 × Ftd − 20.320	–
**12.0**	T = 0.2137 × Ftd − 4.8349	–
**24.0**	T = 0.1810 × Ftd − 1.3526	–
**48.0**	T = 0.1031 × Ftd − 14.277	–

## Data Availability

Data sharing not available.
